# Germline mutations in the oncogene EZH2 cause Weaver syndrome and increased human height

**DOI:** 10.18632/oncotarget.385

**Published:** 2011-12-21

**Authors:** Katrina Tatton-Brown, Sandra Hanks, Elise Ruark, Anna Zachariou, Silvana Del Vecchio Duarte, Emma Ramsay, Katie Snape, Anne Murray, Elizabeth R Perdeaux, Sheila Seal, Chey Loveday, Siddharth Banka, Carol Clericuzio, Frances Flinter, Alex Magee, Vivienne McConnell, Michael Patton, Wolfgang Raith, Julia Rankin, Miranda Splitt, Volker Strenger, Clare Taylor, Patricia Wheeler, I Karen Temple, Trevor Cole, The Childhood Overgrowth Collaboration, Jenny Douglas, Nazneen Rahman

**Affiliations:** ^1^ Division of Genetics & Epidemiology, Institute of Cancer Research, Sutton, UK; ^2^ Medical Genetics, St George's University of London, London, UK; ^3^ Genetic Medicine, University of Manchester, Manchester, UK; ^4^ Pediatric Genetics, University of New Mexico, Albuquerque, USA; ^5^ Clinical Genetics, Guy's and St Thomas' Foundation Trust, London, UK; ^6^ Northern Ireland Regional Genetics Service, Belfast City Hospital, Belfast, Northern Ireland, UK; ^7^ Division of Neonatology, Department of Paediatrics, Medical University, Graz, Austria; ^8^ Peninsula Clinical Genetics Service, Royal Devon and Exeter Foundation NHS Trust, Exeter, UK; ^9^ Institute of Human Genetics, International Centre for Life, Newcastle upon Tyne, UK; ^10^ Department of Paediatrics and Adolescent Medicine, Medical University of Graz, Graz, Austria; ^11^ Institute of Medical Genetics, University Hospital of Wales, Cardiff, UK; ^12^ Division of Genetics, Nemours Children's Clinic, Orlando, USA; ^13^ Human Genetics and Genomic Medicine, Faculty of Medicine, University of Southampton, Southampton, UK; ^14^ West Midlands Regional Genetics Service, Birmingham Women's Hospital, Birmingham, UK; ^15^A full list of members appears in the Supplementary Appendix

**Keywords:** EZH2, Weaver syndrome, height, myeloid malignancies, histone methyltransferase

## Abstract

The biological processes controlling human growth are diverse, complex and poorly understood. Genetic factors are important and human height has been shown to be a highly polygenic trait to which common and rare genetic variation contributes. Weaver syndrome is a human overgrowth condition characterised by tall stature, dysmorphic facial features, learning disability and variable additional features. We performed exome sequencing in four individuals with Weaver syndrome, identifying a mutation in the histone methyltransferase, *EZH2*, in each case. Sequencing of *EZH2* in additional individuals with overgrowth identified a further 15 mutations. The *EZH2* mutation spectrum in Weaver syndrome shows considerable overlap with the inactivating somatic *EZH2* mutations recently reported in myeloid malignancies. Our data establish *EZH2* mutations as the cause of Weaver syndrome and provide further links between histone modifications and regulation of human growth.

## INTRODUCTION

The control of human growth is a complex process involving multiple different biological pathways. Several conditions associated with human overgrowth are recognised and the underlying causes are extremely diverse [6]. More recently, GWAS studies have identified over 180 loci that contribute to human height, some of which overlap with Mendelian syndromes [1]. Weaver syndrome was first described in 1974 and is characterised by pre and postnatal overgrowth, variable learning disability and a distinctive facial appearance [2, 3]. The majority of cases are sporadic, though rare familial cases exhibiting an autosomal dominant pattern of inheritance have been reported [7-9]. Here we have undertaken exome sequencing and sanger sequencing to identify the cause of Weaver syndrome and to characterise the molecular and clinical associations of the causative gene.

## RESULTS

To identify the cause of Weaver syndrome we first undertook exome capture and sequencing in four individuals with classic features of the condition, using the Agilent SureSelect Human All Exon kits and Illumina GAIIx platform (cases 3, 10, 14 and 16, Table 1). We used NextGENe software to detect sequence variants as previously described [10]. To prioritise variants for consideration we first evaluated only variants with coverage of at least 15 reads and we excluded variants that were intronic, synonymous, recorded in dbSNP and/or were present in 45 exomes we performed in other conditions. After these filters, 1,357 variants remained. We applied a script to ascertain genes with variants in all four individuals. This identified only one gene, *EZH2*, with each case carrying a different *EZH2* mutation (Table 1). We confirmed the four mutations by Sanger sequencing and also showed that the mutations were not present in the seven parental samples available for study, establishing that the mutations had arisen *de novo* in at least three of the individuals with Weaver syndrome.

**Table 1:** Summary of *EZH2* mutations and associated clinical features

Case ID	Mutation; protein alteration	Inheritance	Height^a^ (SD)	OFC^a^ (SD)	Learning disability	Malignancy
**1**	c.401T>C;p.M134T	de novo	+2.2	+0.3	mild	
**2**	c.466A>G;p.K156E	familial	+3.3	+1.5	mild	
**3**	c.836A>G;p.H279R	de novo	+2.6	+2.3	mild	
**4**	c.1876G>A;p.V626M	de novo	+3.6	+3.2	no	
**5**	c.1915A>G;p.K639E	de novo	+6	+4.9	moderate	
**6**	c.1987T>A;p.Y663N	de novo	+5	+3	mild	
**7**	c.1991A>T;p.D664V	nk	+3.8	+3.2	moderate	
**8**	c.2044G>A;p.A682T	de novo	+4.2	+1.8	mild	Neuroblastoma ALL
**9**	c.2050C>T;p.R684C	de novo	+2.9	nk	mild	
**10**	c.2050C>T;p.R684C	nk	+3.5	+1.5	nk	
**11**	c.2050C>T;p.R684C	de novo	+3	+2.6	mild	
**12**	c.2050C>T;p.R684C	nk	+5.6	+1.7	mild	
**13**	c.2084C>T;p.S695L	de novo	+5	nk	mild	
**14**	c.2199C>G;p.Y733X	de novo	+7.6	+2.7	no	
**15**	c.2196-15_2196-2delTTCCTGTTGTTTCA	nk	+4.6	nk	nk	
**16**	c.2204_2211dupAGGCTGAT	de novo	+4.6	+2.2	moderate	
**17**	c.2230_2232dupATC	nk	+3.4	+1.7	mild	
**18**	c.2222A>G;p.Y741C	de novo	+5.3	+2	no	
**19**	c.2233G>A;p.E745K	nk	+3.5	+1.4	mild	Lymphoma

a The height and OFC measurements are the standard deviations relative to the mean

OFC, occipito-frontal circumference; SD, standard deviations; nk, not known; ALL, acute lymphoblastic leukaemia

To further evaluate the role of *EZH2* in human overgrowth conditions we sequenced the full coding sequence and intron-exon boundaries of the gene by Sanger sequencing in an additional 300 individuals (Supplementary Table 1). These cases either had a clinical diagnosis of Weaver syndrome, or a non-specific overgrowth syndrome which we defined as height and/or head circumference at least two standard deviations above the mean, together with variable additional phenotypic features.

**Figure 1:**
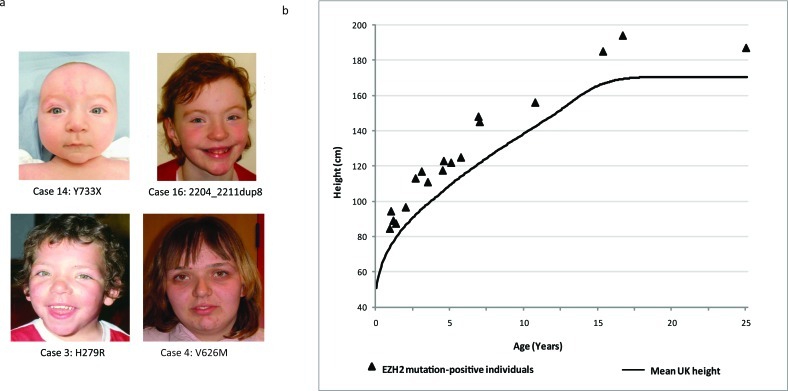
Facial features and height of *EZH2* mutation-positive individuals (a) Typical facial appearance of children with an *EZH2* mutation and Weaver syndrome. (b) Height distribution of *EZH2* mutation-positive individuals relative to the mean UK1990 height [22].

**Figure 2:**
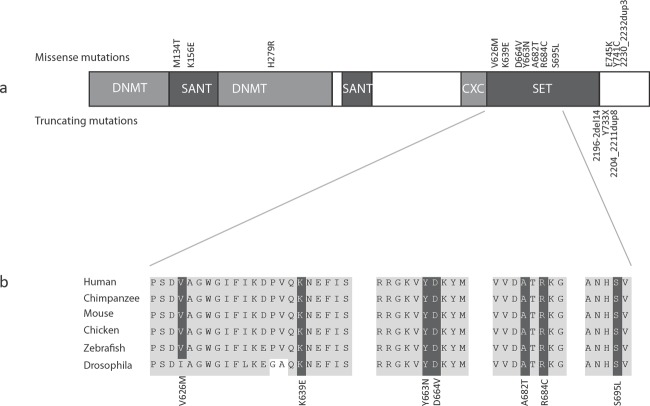
EZH2 structure, mutations and homology (a) Schematic representation of the protein structure of EZH2 with domains and mutations. Missense mutations are represented above the protein and truncating mutations below the protein. (b) Across species homology of the EZH2 SET domain demonstrating missense mutations targeting conserved residues.

We identified mutations that we consider pathogenic in a further 15 individuals (Table 1, Figure 1, Figure 2, Supplementary Table 2). For nine individuals, analysis of parental samples demonstrated that the relevant mutation had arisen *de novo*, confirming pathogenicity. Case 2 had a family history of overgrowth and there was full segregation of the *EZH2* mutation, K156E, with the overgrowth phenotype (Supplementary Figure 1). We identified one recurrent mutation, R684C, which was detected in four unrelated individuals. We were able to demonstrate that at least two had arisen *de novo* and therefore independently. This suggests that R684 is a mutational hotspot and likely reflects the increased mutability of the CpG site at this position. We believe the mutations 2230_2232dupATC, D664V, and E745K are pathogenic as they occur at highly conserved residues and were identified in individuals with a clinical diagnosis of Weaver syndrome. Parental samples were not available and thus it is possible that these are rare polymorphisms. However, analysis of 115 population controls through the full *EZH2* sequence did not reveal either these, or any similar mutations, lending further support that they are pathogenic. The full list of *EZH2* sequence variants we identified is given in Supplementary Table 2.

## DISCUSSION

*EZH2* (Enhancer of Zeste, Drosophila, homolog 2) encodes the catalytic component of the polycomb repressive complex 2 (PRC2), which epigenetically regulates chromatin structure and gene expression through trimethylation at H3K27 and recruitment of DNA methyltransferases, both of which act to repress transcription [11, 12]. EZH2 also has critical roles in stem cell maintenance and cell lineage determination, including osteogenesis, myogenesis, lymphopoiesis and hematopoiesis [13]. EZH2 contains a number of functional domains and it is noteworthy that the majority of the mutations we identified are missense mutations that target highly conserved residues within these domains (Figure 2).

Somatic gain-of-function and loss-of-function mutations in *EZH2* have been reported in haematological malignancies [4, 5, 14, 15]. Activating somatic mutations at Y646, which increase H3K27 trimethylation, have been reported in 22% of diffuse large-cell B-cell lymphomas and in 7% of follicular lymphomas [14, 16]. Inactivating somatic *EZH2* mutations have been reported in myeloid neoplasms such as poor prognosis myelodysplasia-myeloproliferative neoplasms (10-13%), myelofibrosis (13%) and various subtypes of myelodysplastic syndromes (6%) [4, 5, 15].

The mutations we identified in Weaver syndrome show considerable overlap with the mutations in myeloid malignancies. Indeed, three of the mutations, D664V, R684C and Y733X have also been detected as somatically acquired mutations in CMML and myelofibrosis [4]. Only two of the *EZH2* mutation-positive individuals we report have developed malignancies; case 8 who carries a *de novo* missense mutation, A682T, in the SET domain, developed neuroblastoma and acute lymphoblastic lymphoma, both diagnosed at 13 months. Case 19 harbours a missense mutation, E745K, in the post-SET domain and developed lymphoma at 12 years. However, it should be noted that the oldest mutation-positive individual for whom we have follow up data is only 27 years old, and little long-term clinical data is currently available in Weaver syndrome. Given that the myeloid malignancies associated with somatic *EZH2* mutations usually occur in later life it is possible that individuals with Weaver syndrome are at increased risk of myeloid and/or other malignancies.

Although there is overlap of the mutational spectra in Weaver syndrome and myeloid malignancies, there are also differences. The majority of mutations we identified are missense variants. We identified only three truncating mutations; all affect the last *EZH2* exon and therefore may not initiate nonsense mediated RNA decay. By contrast, somatic truncating mutations have been reported throughout *EZH2* [4, 5]. Additionally, germline mutations that result in full gene inactivation are predominantly truncating mutations that occur throughout the gene, for example *NSD1* mutations in the overgrowth condition, Sotos syndrome [17]. These data suggest that the mutations in Weaver syndrome are not acting simply by generating haploinsufficiency. Larger, long-term studies of Weaver syndrome, together with functional analyses, should provide further insights into the nature of causative mutations and associated cancer risks.

There were consistent clinical features amongst the 19 individuals with *EZH2* mutations. Most prominent was increased height. The height of all mutation-positive individuals was at least two standard deviations above the mean and in nine individuals it was greater than four standard deviations above the mean (Table 1, Figure 1). Increase in head circumference was less dramatic, in contrast to Sotos syndrome and the *PTEN*-associated syndromes in which macrocephaly is the more prominent and consistent manifestation of overgrowth (Table 1) [6, 17, 18]. Learning disability was frequent and usually in the mild-moderate spectrum, although some individuals had no reported learning difficulties. *EZH2* mutation-positive individuals have a recognisable facial phenotype that includes a round face, high broad forehead, high hairline and hypertelorism (Figure 1). Although distinctive, indeed 16 of the 19 individuals we identified were referred with a clinical diagnosis of Weaver syndrome, overall the clinical characteristics can be relatively subtle and there may be considerable under-diagnosis of this condition.

*EZH2* is the second histone methyltransferase associated with human overgrowth. Weaver syndrome shares many clinical similarities with the overgrowth condition, Sotos syndrome, which is due to mutations in the histone methyltransferase *NSD1* [17]. Moreover, EZH2 is part of the PI3K/mTOR pathway which includes other genes that have been associated with dysregulated growth in humans [19]. For example, a somatic mutation in *AKT1* causes Proteus syndrome [20] and germline *PTEN* mutations can cause macrocephaly and overgrowth [6, 18]. It is also of interest that these pathways were not strongly implicated in large-scale, genome-wide association studies of height [1]. This suggests that common variation in these genes may not contribute to human growth regulation and further emphasises the importance of studies of rare genetic syndromes in the explication of fundamental biological processes.

## METHODS

### Samples

#### Cases

Individuals with overgrowth were recruited through the Childhood Overgrowth Study. Informed consent was obtained from all participants and the research had approval from the London Multicentre Ethics Committee, (Reference: MREC/01/2/44). A full list of collaborators is given in the Supplementary Appendix. Cases were included in the *EZH2* mutation screening if they had a clinical diagnosis of Weaver syndrome or if they had a non-specific overgrowth phenotype. Included amongst the individuals with Weaver syndrome were five who had previously been reported, cases 3, 5, 6, 7, and 17 [3, 21]. The group with non-specific overgrowth included individuals with global overgrowth, where both height and head circumference were at least two standard deviations above the mean, individuals with isolated macrocephaly where the head circumference was at least two standard deviations above the mean but height was below two standard deviations above the mean and individuals with height but not head circumference greater than two standard deviations above the mean. For figure 1, the height of *EZH2* mutation-positive individuals was plotted with reference to the mean height which was calculated using sex-averaged data from male and female UK1990 growth charts, which was provided by the Child Growth Foundation [22].

#### Controls

DNA samples from UK controls were from the 1958 Birth Cohort, an ongoing follow-up of persons born in Great Britain in one week in 1958 which included a biomedical assessment during 2002-2004 at which blood samples and informed consent were obtained for creation of a genetic resource http://www.cls.ioe.ac.uk/studies.asp?section=000100020003.

### Exome sequencing

The exome preparation and sequencing for cases 3, 10 and 16 was performed at Ambry Genetics Corp. (Aliso Viejo,CA, USA) using the Agilent SureSelect Human All Exon 38 Mb Kit. Each sample was run on two lanes of an Illumina Genome Analyzer IIx generating 2×76 bp reads. We performed the exome sequencing for case 14 in-house using the Illumina Genomic PE Sample Prep Kit (Ilumina, San Diego, CA, USA) and the Agilent SureSelect Human All Exon 50 Mb Kit. This sample was run on two lanes of an Illumina HiSeq Analyzer generating 2×100 bp reads.

### Exome sequence analysis

We identified variants in the exomic sequence using NextGENe software version 2.10 (SoftGenetics, State College, PA, USA). We excluded variants that were intronic, synonymous, detected in a known pseudogene, recorded in dbSNP or detected in 45 in-house exomes from individuals with familial breast cancer that we have performed as part of a separate study. To prioritise evaluation of variants most likely to be real we first included only variants with coverage of at least 15 reads. In this final variant set we ran a script to identify genes with variants in all four individuals.

### *EZH2* mutation analysis

We performed Sanger sequencing of PCR products from genomic DNA to confirm the mutations identified by exome sequencing, and to mutationally analyse the full coding sequence in the overgrowth series. We designed PCR primers to amplify the 19 coding exons and intron-exon boundaries of *EZH2* in 3 multiplex PCR reactions (Supplementary Table 1). The PCR was carried out using a Qiagen Multiplex PCR kit according to the manufacturer's instructions. Products were sequenced with the original PCR primers or internal sequencing primers (exons 3 and 20) using the BigDye Terminator Cycle Sequencing Kit and an ABI 3730 Genetic Analyzer (Applied Biosystems, Foster City, CA,USA). Sequences were analyzed using Mutation Surveyor software v3.97 (SoftGenetics, State College, PA, USA). All mutations were confirmed by bidirectional sequencing of a second, independently amplified PCR product.

### *In silico* analyses of identified variants

We computed the predicted effects of *EZH2* nonsynonymous variants on protein function using PolyPhen [23] and SIFT [24]. All variants (intronic and coding) were analysed for their potential effect on splicing. Variants were analysed using two splice prediction algorithms NNsplice [25] and MaxEntScan, [26] via the Alamut software interface (Interactive Biosoftware). If both NNsplice and MaxEntScan scores were altered by >20% (i.e. a wildtype splice-site score decreases and/or a cryptic splice-site score increases) three further prediction algorithms were utilised; NetGene2 [27], HumanSplicingFinder [28] and Genscan [29]. A consensus decrease in a wildtype splice-site score and/or a consensus increase in a cryptic splice-site score across all algorithms was considered indicative of disruption of normal splicing. To evaluate the conservation of variants we used the HomoloGene system for automated detection of homologs among the annotated genes of completely sequenced eukaryotic genomes. http://www.ncbi.nlm.nih.gov/homologene/37926.

### Accession codes

*EZH2* mutation nomenclature corresponds to Ensembl Transcript ID ENST00000337432.

### URLs

dbSNP: ncbi.nlm.nih.gov/projects/SNP/

HomoloGene: ncbi.nlm.nih.gov/homologene/37926.

Mutation Surveyor and NextGENe software: softgenetics.com

## Supplementary Figures, Tables and Appendix


