# Correction: Germline mutations in the oncogene EZH2 cause Weaver syndrome and increased human height

**DOI:** 10.18632/oncotarget.26429

**Published:** 2018-11-30

**Authors:** Katrina Tatton-Brown, Sandra Hanks, Elise Ruark, Anna Zachariou, Silvana Del Vecchio Duarte, Emma Ramsay, Katie Snape, Anne Murray, Elizabeth R Perdeaux, Sheila Seal, Chey Loveday, Siddharth Banka, Carol Clericuzio, Frances Flinter, Alex Magee, Vivienne McConnell, Michael Patton, Wolfgang Raith, Julia Rankin, Miranda Splitt, Volker Strenger, Clare Taylor, Patricia Wheeler, I Karen Temple, Trevor Cole, The Childhood Overgrowth Collaboration, Jenny Douglas, Nazneen Rahman

**Affiliations:** ^1^ Division of Genetics & Epidemiology, Institute of Cancer Research, Sutton, UK; ^2^ Medical Genetics, St George's University of London, London, UK; ^3^ Genetic Medicine, University of Manchester, Manchester, UK; ^4^ Pediatric Genetics, University of New Mexico, Albuquerque, USA; ^5^ Clinical Genetics, Guy's and St Thomas' Foundation Trust, London, UK; ^6^ Northern Ireland Regional Genetics Service, Belfast City Hospital, Belfast, Northern Ireland, UK; ^7^ Division of Neonatology, Department of Paediatrics, Medical University, Graz, Austria; ^8^ Peninsula Clinical Genetics Service, Royal Devon and Exeter Foundation NHS Trust, Exeter, UK; ^9^ Institute of Human Genetics, International Centre for Life, Newcastle upon Tyne, UK; ^10^ Department of Paediatrics and Adolescent Medicine, Medical University of Graz, Graz, Austria; ^11^ Institute of Medical Genetics, University Hospital of Wales, Cardiff, UK; ^12^ Division of Genetics, Nemours Children's Clinic, Orlando, USA; ^13^ Human Genetics and Genomic Medicine, Faculty of Medicine, University of Southampton, Southampton, UK; ^14^ West Midlands Regional Genetics Service, Birmingham Women's Hospital, Birmingham, UK; ^15^ A full list of members appears in the Supplementary Appendix

**This article has been corrected:** In the supplementary file on page 4, the corrected author name is given below:

**M. Tischkowitz**

Original article: Oncotarget. 2011; 2:1127-1133. https://doi.org/10.18632/oncotarget.385

