# Supplementary material for: Germline mutations in the oncogene EZH2 cause Weaver syndrome and increased human height
Source: Oncotarget. 2011 Dec 21;2(12):1127–33. doi: 10.18632/oncotarget.385 (PMC3282071; doi:10.18632/oncotarget.385)
Supplement: Supplementary file 1 [file oncotarget-02-1127-s001.pdf]

Germline mutations in the oncogene *EZH2* cause Weaver syndrome and increased human height

Katrina Tatton-Brown[1,2], Sandra Hanks[1], Elise Ruark[1], Anna Zachariou[1], Silvana Del Vecchio Duarte[1], Emma Ramsay[1], Katie Snape[1], Anne Murray[1], Elizabeth R Perdeaux[1], Sheila Seal[1], Chey Loveday[1], Siddharth Banka[3], Carol Clericuzio[4], Frances Flinter[5], Alex Magee[6], Vivienne McConnell[6], Michael Patton[2], Wolfgang Raith[7], Julia Rankin[8], Miranda Splitt[9], Volker Strenger[10], Clare Taylor[11], Patricia Wheeler[12], I Karen Temple[13], Trevor Cole[14], The Childhood Overgrowth Collaboration[15], Jenny Douglas[1] and Nazneen Rahman[1*]

Supplementary Tables

Supplementary Table 1 Primers for *EZH2* mutation analysis

Exon	PCR Primer Forward	PCR Primer Reverse	Size (bp)	Multi-plex Group
2	TGATTGTTAGTTTGCTGCGG	CTAAGCTTCCAAGTATTCACTCATC	353	A
3[a]	TTTTGTATTATTTGAATGTGGGAAAC	AAGATGGACACCCTGAGGTC	408	B
4	ACCCTAAGTAAAAGAAAAGAGAGAATC	GGAAAAGAGTAATACTGCACAGG	488	C
5	AAATCTGGAGAACTGGGTAAAGAC	GCCCAGGTTCAGTCCCTTATAG	375	A
6	AGGCTATGCCTGTTTTGTCC	AAAAGAGAAAGAAGAAACTAAGCCC	332	B
7	CTGACTGGCATTCCACAGAC	AAGTGTAGTGGCTCATCCGC	380	C
8	GCCATTCCTTTATGTTTTAGGC	GCAAAAGATTTCAGAGCAATCC	482	A
9	GGAAAGTTGTATGAAGTAACGTAAACC	GGTGCAGACAACATTAACGC	323	B
10	TTCACAATATAGAACTGTCTTGCC	TGGTCTTTATACTGAAACTAACCAAC	614	C
11	GATGGGTTAGTGTTTTGCCG	TCATTCAATTCCCTCTTGGG	458	B
12	ATCCCCAGCATCTAGCAGTG	TTCCCAGTGGAAATAAGATTCC	374	C
13	TCTTGGCTTTAACGCATTCC	CAAATTGGTTTAACATACAGAAGGC	289	A
14	AGGCCAGCTACACTCCACAG	AGGGAGTGCTCCCATGTTC	333	B
15	GAGAGTCAGTGAGATGCCCAG	TTTGCCCCAGCTAAATCATC	371	C
16	TGTGCCCAATTACTGCCTTAG	TTTCCAATCAAACCCACAGAC	334	A
17	GCGTTTTCTCCAGAAGGTCC	TCTGGTCACCTCACTGACCTC	292	B
18	TGAAGCTGCTTGATTTATTTGC	TGGTGTCAGTGAGCATGAAG	355	C
19	GATGGACTTGAATACTTCTGGG	TTCCCCACTAATGCTCATGG	320	A
20[b]	ATGAATGTGCCGTTATGCAG	AAAACACTTTGCAGCTGGTG	422	B

[a]Sequencing primer used: CTCCCAATAACCAAACAA
[b]Sequencing primer used: AAGTCTCAGCACATGTTG
The forward primer was used for sequencing all other exons except exons 10, 11 and 17 for which the reverse PCR primer was used.

Supplementary Table 2 *EZH2* sequence variants

Mutation	Case / control	rs number	SIFT	Polyphen	Consensus splice prediction
**Common variants**					
c.396T>C;p.P132P	both	rs61732845	na	na	benign
c.553G>C;p.D185H	both	rs52833659	deleterious	benign	benign
c.1731G>A;p.P577P	both	rs41277437	na	na	benign
c.2110+6T>G	both	rs41277434	na	na	benign
**Rare variants**					
c.401T>C;p.M134T	1 case		deleterious	possibly damaging	benign
c.466A>G;p.K156E	1 case		tolerated	probably damaging	benign
c.698A>T;p.D233V	1 case*		deleterious	probably damaging	benign
c.836A>G;p.H279R	1 case		tolerated	probably damaging	benign
c.965A>G;p.N322S	1 control		tolerated	benign	benign
c.1147A>G;p.T383A	1 case**		tolerated	benign	benign
c.1458C>T;p.P486P	2 cases, 1 control		na	na	benign
c.1459G>A;p. A487T	1 case		tolerated	benign	benign
c.1876G>A;p.V626M	1 case		deleterious	probably damaging	benign
c.1915A>G;p.K639E	1 case		deleterious	probably damaging	benign
c.1947G>A;p.E649E	1 control		na	na	benign
c.1987T>A;p.Y663N	1 case		deleterious	probably damaging	benign
c.1991A>T;p.D664V	1 case		deleterious	probably damaging	benign
c.2044G>A;p.A682T	1 case		deleterious	probably damaging	benign
c.2050C>T;p.R684C	4 cases		deleterious	probably damaging	benign
c.2084C>T;p.S695L	1 case		deleterious	probably damaging	benign
c.2199C>G;p.Y733X	1 case		na	na	benign
c.2196-15_2196-2del14	1 case		na	na	disrupts splicing
c.2204_2211dup8	1 case		na	na	benign
c.2230_2232dupATC	1 case		na	na	benign
c.2222A>G;p.Y741C	1 case		deleterious	probably damaging	benign
c.2233G>A;p.E745K	1 case		tolerated	probably damaging	benign

*Present in father, phenotype unknown,
** Present in unaffected father
na, not applicable

Supplementary Figures

Supplementary Figure 1 Pedigree of case 2.

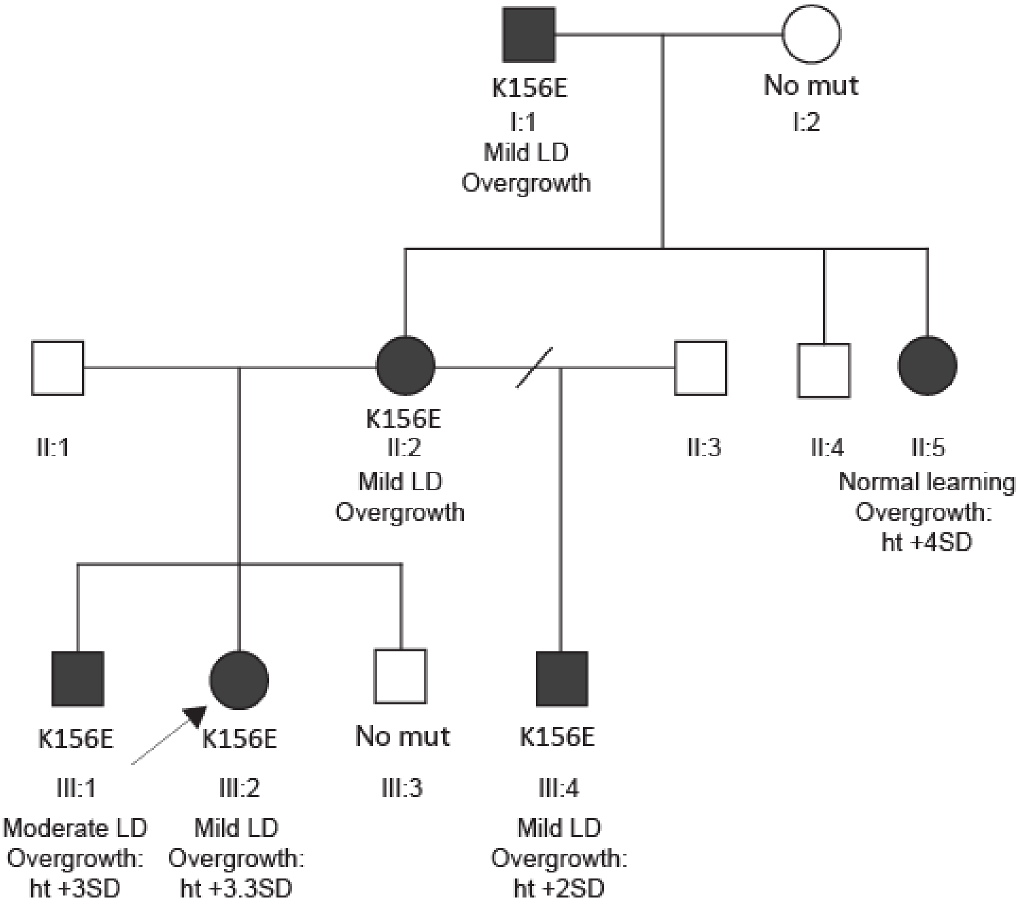

Affected individuals are shown as shaded symbols and unaffected individuals as unshaded symbols. The proband is indicated with an arrow and corresponds to the case 2 individuals in Table 1.The mutation status of the individual is shown below the symbol along with relevant clinical information. LD, learning disability; ht, height.

Supplementary Appendix

The Childhood Overgrowth (COG) Collaboration

The following are members of the Childhood Overgrowth Collaboration and undertook recruitment of cases, samples and clinical information from individuals/families with childhood overgrowth.

D. Amor, S. Andries, H. Archer, R. Armstrong, P. Ashton-Prolla, D. Baralle, A. Barnicoat, M. Barrow, P. Beales, K. Becker, E. Beckh-Arnold, J. Berg, B. Bernhard, M. Bhat, J. Birch, M. Bitner, E. Blair, J. Bliek, M. Blyth, A. Brady, G. Brice, L. Brueton, J. Burn, N. Canham, B. Castle, M. Cecconi, K. Chandler, R. Chandrasena, D. Cilliers, A. Clarke, J. Clayton-Smith, C. Clericuzio, T. Cole, A. Colley, A. Collins, F. Connell, J. Cook, Y. Crow, T. Dabir, A. Dalton, S. Danda, S. Davies, R. Day, N. Dennis, C. Deshpande, B. Desouza, L. Devlin, A-M. Differ, R. Dinwiddie, A. Dobbie, D. Donnai, I. Ellis, F. Elmslie, H. Firth, R. Fisher, D. Fitzpatrick, F. Flinter, P. Foley, N. Foulds, A. Fryer, A. Gallagher, S. Garcia, C. Gardiner, R. Gibbons, Y. Gillerot, D. Goudie, K. Gowrishanker, C. Graham, N. Gregersen, J. Harper, H. Hughes, A. Henderson, R. Hennekam, E. Hobson, S. Holder, T. Homfray, Z. Huma, J. Hurst, M. Irving, L. Izatt, S. Jagadeeth, C. Jessen, D. Johnson, D. Josifova, S. Joss, B. Kerr, J. Liebelt, U. Kini, A. Krause, A. Kumar, D. Kumar, W. Lam, P. Lapunzina, M. Lees, N. Leonard, A. Livesey, C. Longman, A. Lucassen, P. Lunt, S. Lynch, J. MacDonnell, A. Magee, E. Maher, A. Male, S. Mansour, V. McConnell, M. McEntagart, S. McKee, C. McKeown, , S. Mehta, K. Metcalfe, S. Mohammed, G. Monaghan, T. Montgomery, A. Morgan, P. Morrison , J. Morton, R. Mudgal, V. Murday, S. Nampoothiri, A. Nemeth, R. Newbury-Ecob, C. Oley, C. Owen, S-M. Park, M. Parker, C. Patel, M. Patton, D. Pilz, M. Pinkney, M. Pocha, C. Pottinger, K. Prescott, S. Price, A. Proctor, O. Quarrell, J. Rankin, L. Raymond, G. Rea, W. Reardon, E. Reid, M. Robards, A. Roposch, E. Rosser, D. Rourke, D. Ruddy, A. Saggar, J. Sampson, R. Sandford, A. Sarkar, R. Scott, R. Semple, S. Sharif, A. Shaw, C. Shaw-Smith, D. Shears, J. Shelagh, G. Smith, S. Smithson, M. Splitt, M. Stevens, F. Stewart, H. Stewart, K. Stopps, M. Suri, E. Sweeney, G. Tanateles, C. Taylor, K. Temple, M. Tischowitz, J. Tolmie, S. Tomkins, P. Turnpenny, M. Van-Haelst, L. Van Maldergem, A. Vandersteen, P. Vasudevan, E. Wakeling, L. Walker, D. Williams, L. Wilson, G. Woods, M. Wright, A. Zankl
